# Assessment of needs-based usefulness of interventions to address system barriers to HPV vaccination

**DOI:** 10.1093/eurpub/ckac130.005

**Published:** 2022-10-25

**Authors:** T Schloemer, H Hecht, K Horstman

**Affiliations:** 1 Care and Public Health Research Institute, Maastricht University, Maastricht, Netherlands; 2 Department of Applied Health Sciences, Hochschule für Gesundheit, Bochum, Germany

## Abstract

**Background:**

Adolescents of underserved communities face multiple health system barriers to access HPV vaccination. The Horizon 2020 project RIVER-EU will implement interventions to address these barriers in the migrant community in Greece, the Turkish and Moroccan communities in the Netherlands, the Ukrainian community in Poland and the Roma community in Slovakia. The objective of this study was to identify promising evidence-based interventions that potentially meet the specific contextual needs of the target groups, as a basis for assessing their transferability.

**Methods:**

Based on the PIET-T models of transferability, we developed a methodology to assess the needs-based usefulness of an intervention for a target context. Criteria of intervention usefulness addressed specific aspects of the health issue, population, intervention content, outcomes, up-to-datedness, applicability, quality and usefulness of the evidence-base. Guided by methodological workshops, country members with their local advisory boards performed a rating of 32 interventions identified from a systematic literature search.

**Results:**

Through independent assessment in each country, 5 of 32 interventions were selected as useful, with overlap: 2 interventions were selected by all countries, and 1 by 2 countries. In all countries, trained community members to support HPV vaccination were seen as promising. In Greece and the Netherlands, an educational programme in schools was included. Further, the Netherlands chose an intervention addressing providers’ HPV vaccine communication, and Slovakia a multilevel intervention.

**Discussion:**

The feedback of the country members on the assessment emphasised the structured way to face complexity of understanding the potential usefulness of an intervention from the perspective of the target country. Critical discussions on the interventions enabled to specify needs for further clarification, for adaptations, and alternatives to consider for transferability analyses.

**Key messages:**

- Identifying potentially useful health system approaches to vaccination in specific contexts is a complex step that goes beyond analysing quality of primary evidence.
- Our methodology for intervention selection emphasises the consideration of the unique needs of target communities to address health system barriers to vaccination.

